# γδ T cells and resistance to CDK4/6 inhibitors in breast cancer

**DOI:** 10.1038/s41419-025-08149-z

**Published:** 2025-11-14

**Authors:** Oliver Kepp, Guido Kroemer

**Affiliations:** 1 Université Paris Cité, Sorbonne Université, Inserm, Centre de Recherche des Cordeliers, Equipe labellisée par la Ligue contre le cancer, Institut Universitaire de France, Paris, France; 2 Université Paris-Saclay, INSERM US23/CNRS UAR 3655, Metabolomics and Cell Biology Platforms, Institut Gustave Roussy, Villejuif, France; 3 Institut du Cancer Paris CARPEM, Department of Biology, Hôpital Européen Georges Pompidou, AP-HP, Paris, France

**Keywords:** Immunosuppression, Outcomes research

Endocrine therapy remains the cornerstone for the treatment of advanced and metastatic hormone-receptor-positive, HER2-negative (HR^+^/HER2^-^) breast cancer. The introduction of cyclin-dependent kinase-4/6 (CDK4/6) inhibitors, palbociclib, ribociclib, and abemaciclib, into clinical practice, has enhanced the clinical benefit of endocrine therapy through the inhibition of retinoblastoma (RB) phosphorylation. This has translated into prolonged progression-free intervals and overall survival [1–3].

Nevertheless, therapeutic pressure inevitably drives the emergence of resistance, limiting the durability of responses. While CDK4/6 inhibitors are best known for their cytostatic activity, increasing evidence suggests they also modulate tumor immunity. These agents can induce a senescence-associated secretory phenotype (SASP), enhance antigen presentation, and selectively deplete regulatory T cells [4, 5]. These immunostimulatory effects have provided a rationale for combining CDK4/6 inhibition with immune-checkpoint blockade in clinical trials.

Paradoxically, however, recent findings suggest that CDK4/6 inhibition can also activate compensatory immunosuppressive circuits within the tumor microenvironment, potentially undermining long-term therapeutic efficacy. Petroni et al., using murine models of HR^+^/HER2^-^ breast cancer, demonstrated that the combination of palbociclib and endocrine therapy provokes a hypoxia-sensitive, chemokine (C-C motif) ligand 2 (CCL2)-mediated influx of interleukin-17A (IL-17A)-secreting γδ T cells. These γδ T lymphocytes reprogram tumor-associated macrophages towards a CX3C motif chemokine receptor 1-positive (CX3CR1^+^) state characterized by reduced antigen presentation and diminished type-I-interferon signaling, thereby fostering resistance [6].

Importantly, depleting γδ T cells or neutralizing IL-17A restored sensitivity to CDK4/6 inhibitors and increased CD8^+^ T cell infiltration [6]. Notably, the administration of radiotherapy prior to palbociclib treatment prevents γδ T cell recruitment by inducing a hypoxic, CCL2-deficient tumor microenvironment [6]. Thus, in this context γδ T cells function as mediators of immune evasion rather than effectors of anti-tumor immunity, which stands in contrast to the cytotoxic or regulatory roles traditionally attributed to them [6, 7].

A similar immunosuppressive role for γδ T cells has been described in the study by Rozalén et al. [8]. Investigating tumor-intrinsic hepatitis A virus cellular receptor 2 (HAVCR2, best known as TIM3), the authors show that breast cancer cells expressing high levels of TIM3 remodel early liver metastases by enriching immunosuppressive myeloid populations and reducing the presence of effector lymphocytes, compared to TIM3-knockdown lesions [8]. This immune reprogramming aligns with previous studies implicating IL-17-producing γδ T cells in neutrophil recruitment, suppression of cytotoxic T cell activity, and promotion of metastatic progression [9].

Together, these findings support a model in which IL-17A^+^ γδ T cells facilitate tumor immune evasion and metastatic outgrowth. In the context of CDK4/6 inhibition, they impair drug efficacy and may contribute to the formation of immune-privileged niches during metastatic seeding (Fig. 1).

**Fig. 1:**
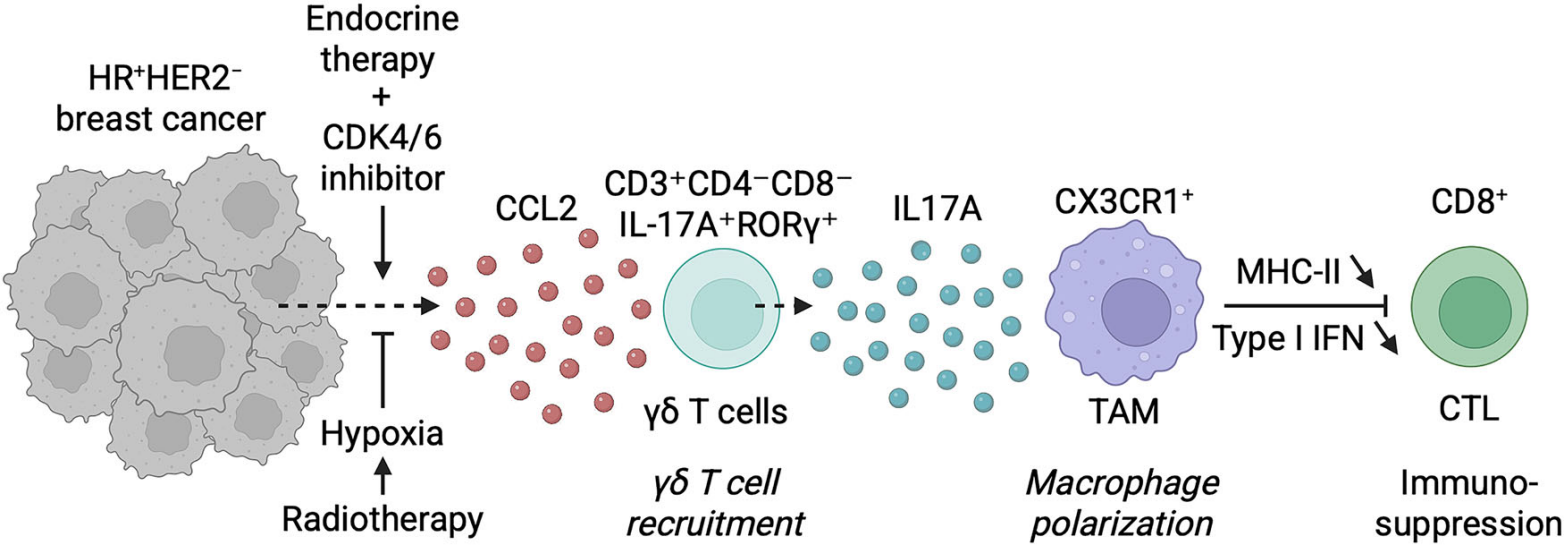
Mechanism of γδ T cell-mediated resistance to CDK4/6 inhibitors in HR^+^HER2^-^ breast cancer. CDK4/6 inhibitor treatment combined with endocrine therapy triggers hypoxia-sensitive cancer cell secretion of the chemokine CCL2. Acting as a chemoattractant, CCL2 recruits IL-17A-producing γδ T cells (CD3⁺CD4⁻CD8⁻IL-17A⁺RORγ⁺) into the tumor microenvironment. These γδ T cells release IL-17A, promoting the repolarization of tumor-associated macrophages (TAMs) toward a CX3CR1⁺ immunosuppressive phenotype. CX3CR1⁺ TAMs display impaired antigen presentation due to downregulated MHC-II expression and weakened type I interferon signaling, fostering an immunosuppressive environment that limits CD8⁺ cytotoxic T lymphocyte (CTL) and activity. This γδ T cell-driven immune evasion ultimately underlies resistance to CDK4/6 inhibition and treatment failure. Notably, radiotherapy administered prior to CDK4/6 inhibition creates a hypoxic tumor microenvironment that directly suppresses CCL2 expression, thereby blocking γδ T cell recruitment.

Recognizing γδ T cells as context-dependent drivers of resistance supports the development of therapeutic strategies aimed at neutralizing IL-17A or otherwise modulating γδ T cell activity [10]. Such approaches may enhance the efficacy of both standard endocrine-CDK4/6 targeted regimens and novel immunotherapeutic combinations in HR^+^/HER2^-^ breast cancer.

## Data Availability

Data sharing is not applicable to this article as no new data were created or analyzed in this study.
